# Usefulness of tissue Doppler-derived atrial electromechanical delay for identifying patients with paroxysmal atrial fibrillation

**DOI:** 10.1186/s12947-020-00205-2

**Published:** 2020-06-22

**Authors:** Kanako Akamatsu, Takahide Ito, Masatoshi Miyamura, Yumiko Kanzaki, Koichi Sohmiya, Masaaki Hoshiga

**Affiliations:** Department of Cardiology, Osaka Medical College, Takatsuki, Osaka 569-8686 Japan

**Keywords:** Atrial fibrillation, Tissue Doppler imaging, Atrial electromechanical delay

## Abstract

**Background:**

Tissue Doppler imaging (TDI)-derived atrial electromechanical delay (AEMD) has been reported to be useful for detecting paroxysmal atrial fibrillation (PAF). However, its usefulness remains unknown when analyzed along with patients seemingly at high-risk for AF as controls. From this standpoint, we investigated whether AEMD would be of use for identifying patients with PAF.

**Methods:**

We retrospectively analyzed TDI recordings to obtain AEMD in 63 PAF patients. Thirty-three patients with multiple cardiovascular risk factors (MRFs) but without history of AF and 50 healthy individuals served as disease and healthy controls, respectively. AEMD was defined as the time-interval between the electrocardiogram P-wave and the beginning of the spectral TDI-derived A’ for the septal (septal EMD) and lateral (lateral EMD) sides of the mitral annulus.

**Results:**

There was no significant difference in the left atrial volume index between PAF patients and disease controls (28 ± 9 mL/m^2^ vs. 27 ± 5 mL/m^2^). PAF patients had longer AEMD, particularly for the lateral EMD (75 ± 23 ms), compared with disease (62 ± 22 ms, *P* = 0.009) and healthy (54 ± 24 ms, *P* < 0.001) controls. Multivariate logistic regression analysis revealed that the lateral EMD (OR 1.25, 95%CI 1.03–1.52, *P* = 0.023), along with the left atrial volume index (OR 2.25, 95%CI 1.44–3.51, *P* < 0.001), was one of the significant independent associates of identifying PAF patients.

**Conclusions:**

This cross-sectional study indicates that even analyzed together with MRFs patients, AEMD remains useful for identifying patients at risk for AF. Our results need to be confirmed by a large-scale prospective study.

## Background

Atrial fibrillation (AF) is one of the most common types of cardiac arrhythmias associated with increased cardiovascular morbidity and mortality. Risk factors of AF include advanced age, male gender, and presence of hypertension [1, 2]. An increased left atrial (LA) volume index is also known as a strong predictor of AF [3–5]. It has been reported that the intra- and interatrial conduction time, that is, the atrial electromechanical delay (AEMD), is an index of reflecting pathological changes of the atria [6–13]. AEMD can be measured not only by invasive electrophysiologic study but also by echocardiographic tissue Doppler imaging (TDI) [7–13]. Previous studies found that TDI-derived AEMD had an advantage to predict AF recurrence over LA diameter and P-wave duration [7]. On the other hand, AEMD was shown to be prolonged in various conditions other than cardiac disorders such as diabetes mellitus and ulcerative colitis [9–12]. However, most of the previous studies regarding AEMD were performed based on the comparison between patients in such diseased conditions and normal controls [9–12]; do not seem to include patients with similar clinical background to AF patients.

In the present study, we retrospectively investigated whether TDI-derived AEMD would be useful to identify patients who had been diagnosed with paroxysmal AF (PAF) in comparison with other variables known as strong predictors of AF including LA volume index. Specifically, this study included patients with multiple cardiovascular risk factors (MRFs) but without history of AF as disease controls in order to test the hypothesis that the ability of AEMD to identify PAF patients was maintained even when patients seemingly at high-risk for AF (i.e., MRFs patients) were included.

## Materials and methods

### Study population

**Fig. 1:**
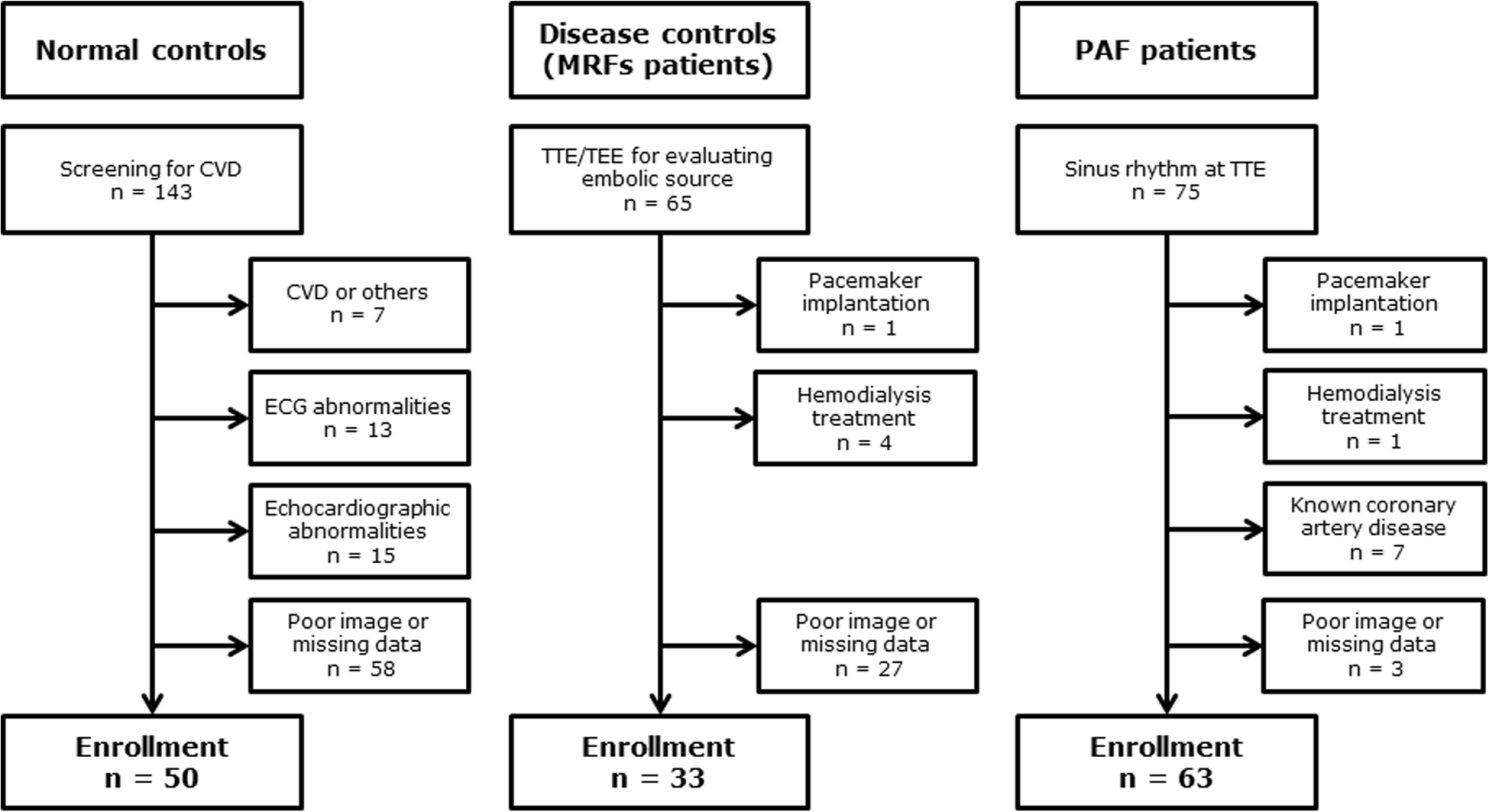
Flowchart for the enrollment of study individuals. CVD, cardiovascular disease; TEE, transesophageal echocardiography; TTE, transthoracic echocardiography

We examined 75 PAF patients and 65 MRFs patients without history of AF, and 143 apparently healthy subjects, all of whom underwent transthoracic echocardiography from February 2012 through December 2018. Patients with previous cardiac surgery including pacemaker implantation, known coronary artery disease, left ventricular (LV) ejection fraction < 30%, and those with dialysis treatment were excluded (Fig. 1). Patients who had echocardiographic images inadequate for assessing indispensable measures, described later, were also excluded.

PAF patients (*n* = 63): All patients were scheduled for pulmonary vein isolation, with their cardiac rhythm being “sinus” during echocardiographic examinations.

Disease controls (MRFs patients) (*n* = 33): This group of patients had been hospitalized in the department of neurology or neurosurgery in our institution under a diagnosis of non-lacunar ischemic stroke, transient ischemic attack, or peripheral artery occlusion. They were found neither to have AF detected on an automated cardiac rhythm monitor [14], nor to have apparent embolic sources detected by carotid ultrasound and transesophageal echocardiography.

Healthy controls (*n* = 50): These individuals were screened for any cardiovascular disease in our outpatient department, and also showed normal results on the routine echocardiographic examination.

This study was approved by the ethics committee in Osaka Medical College with notification for guaranteed withdrawal of participants on the website providing means of “opt-out” (No. 2194–01).

### Standard echocardiography

Transthoracic echocardiography was performed by experienced sonographers using commercially available ultrasound apparatus (Vivid 7 Dimensions or Vivid E9; GE Vingmed Ultrasound, Horten, Norway). During each examination, one-lead electrocardiogram, usually the limb-lead II was recorded continuously. Under 2-dimensional guidance in the parasternal view, LA diameter, LV end-diastolic dimension, and LV wall thickness were measured. LA volume was calculated by the disc method in the apical 2- and 4-chamber views and indexed by the body-surface area leading to LA volume index. LV ejection fraction was measured by the modified Simpson’s rule. LV mass was calculated using the Devereux formula, and indexed by the body surface area (LV mass index). LV mass index ≥115 g/m^2^ in men and ≥ 95 g/m^2^ in women were considered as the presence of LV hypertrophy [15].

For assessing LV diastolic function, pulsed Doppler LV inflow indices of early (E) and late filling (A) wave velocities, their ratio (E/A), and E-wave deceleration time were obtained. In the apical 4-chamber view, using the spectral type of TDI, early (E’) and late (A’) diastolic velocities were measured with the sample volume placed at the septal and lateral sides of the mitral annulus. The ratio of E to E’ (E/E’) was used as a surrogate of LV filling pressure [16]. In the present study, A’, meaning “velocity”, averaged for both mitral annuli, was considered as LA systolic function.

### Measurement of AEMD

**Fig. 2:**
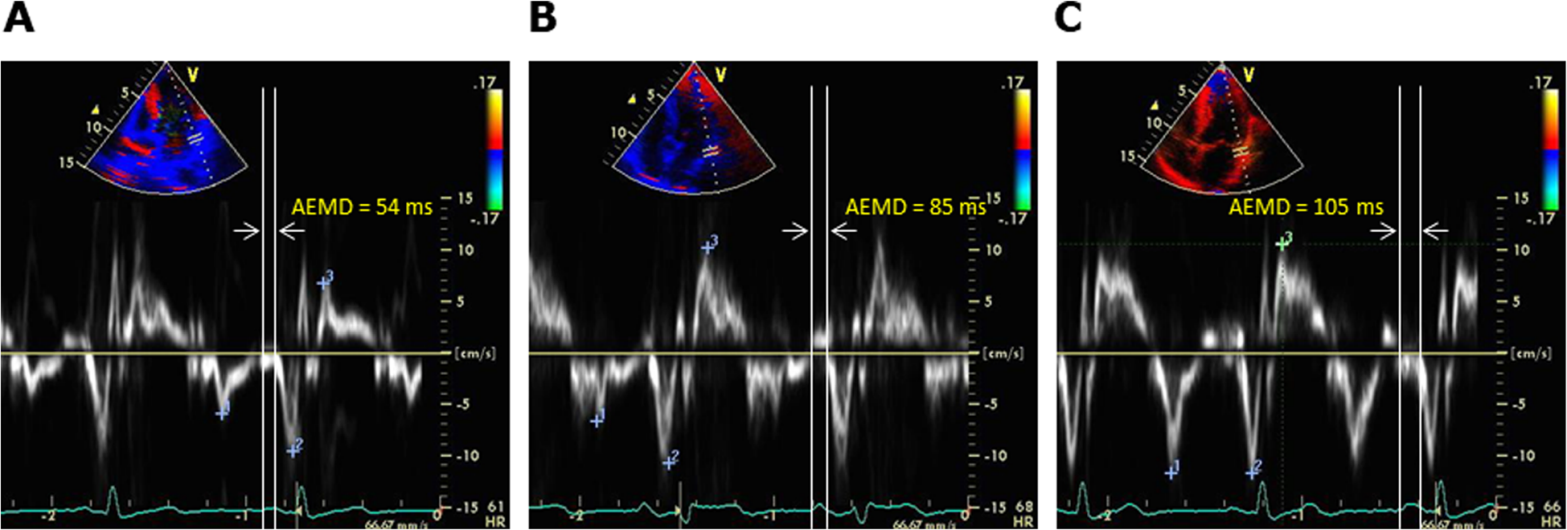
Representative images of explanation how to measure AEMD obtained from: **a** a healthy subject; **b** a patient with multiple cardiovascular risk factors; **c** a PAF patient

AEMD was measured from the beginning of the electrocardiogram P-wave to the initial point of the spectral TDI-derived A’ as described previously [9–12] (Fig. 2). In this study, AEMD was obtained for the septal (septal EMD) and lateral (lateral EMD) sides of the mitral annulus. The time difference of the lateral to septal EMD was defined as intra-LA EMD as reported previously [12]. All AEMD measurements were performed by independent observers without knowledge of patients’ background.

**Fig. 3:**
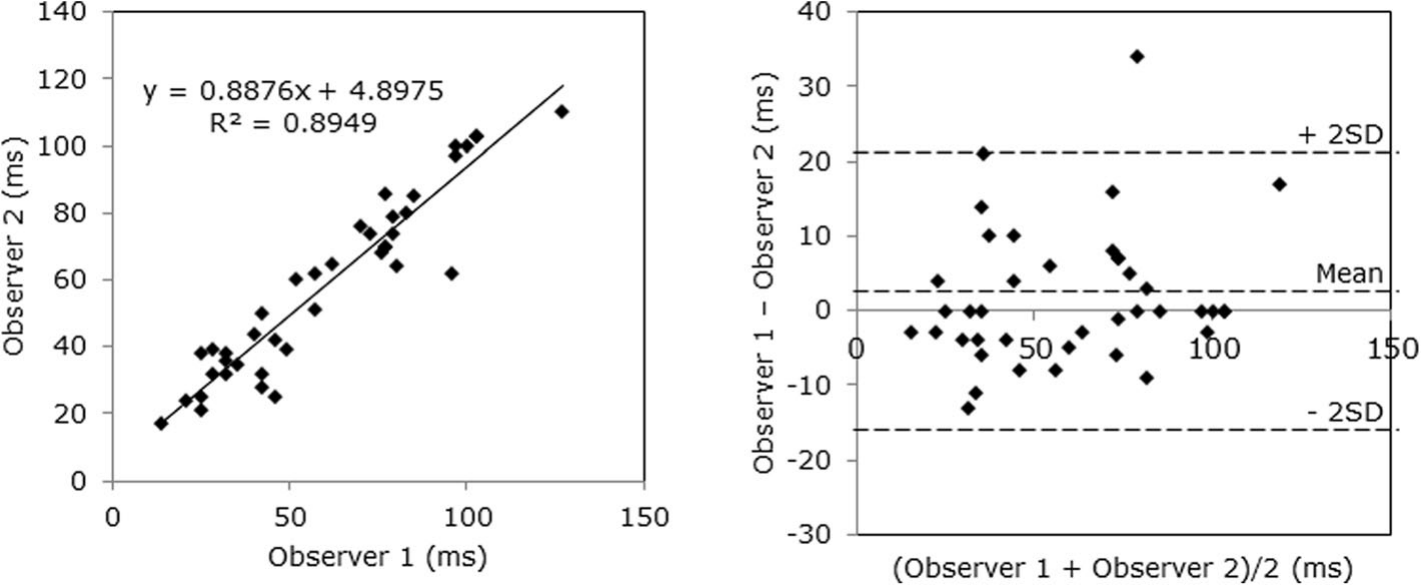
Bland–Altman plot analysis for assessing interobserver variability of AEMD in randomly selected 40 subjects. See the text

To assess interobserver variability of AEMD, 40 individuals were randomly selected and Bland–Altman plot analysis was performed (KA and TI). It was found that measurements were similar and statistically comparable with each other (Fig. 3). The mean difference was 1.9 ms (3.2%) and the coefficient of variation was 4.9.

### Statistical analysis

Continuous variables were expressed as mean ± SD and categorical variables as percentages. Comparisons of categorical variables were made using the chi-square test or Fisher’s exact test. Continuous variables across the 3 groups were compared using one-way analysis of variance or Wilcoxon test according to whether normally distributed or not, as tested by Welch test. Tukey’s HSD test or Wilcoxon Each Pair test was applied for intergroup comparisons as appropriate. Univariate and multivariate logistic regression analysis were performed to predict significant variables for identifying PAF patients. The sensitivity and specificity of AEMD and other echocardiographic variables for identifying PAF patients were calculated by receiver operating characteristic (ROC) analysis. Comparisons of area under curves (AUCs) between models of ROC analysis were also performed. All analyses, except for ROC analysis, were performed using SPSS for Windows ver. 24.0 (IBM, Chicago, IL). For ROC analysis, JMP Pro ver. 13.0 (SAS Institute, Cary, NC) was used. *P* < 0.05 was considered significant.

## Results

### Clinical and echocardiographic data of the study groups

Demographic data of the study groups are summarized in Table 1. Age and gender distribution did not differ among the 3 groups. There was a trend toward increasing body mass index for PAF patients. Disease controls had higher CHADS_2_ and CHA_2_DS_2_-VASc scores compared with PAF patients, primarily as a result of the patient selection. Antiarrhythmic drugs, exclusively being prescribed to PAF patients, included flecainide in 8 patients; verapamil in 4; amiodarone in 5; pilsicainide in 3; and cibenzoline in 1.

**Table 1:** Demographic data of the study groups

Parameters	Healthy controls *(n = 50)*	Disease controls *(n = 33)*	PAF patients *(n = 63)*	*P*
Age (years)	60 ± 14	66 ± 15	62 ± 13	0.21
Female, n (%)	19 (38)	12 (36)	17 (27)	0.41
Body mass index	22.0 ± 3.3	23.1 ± 4.1	24.5 ± 3.6†§	< 0.001
CHADS_2_ score	0.18 ± 0.39	2.88 ± 1.05†	1.27 ± 1.10†‡	< 0.001
CHA_2_DS_2_-VASc score	0.82 ± 0.92	4.15 ± 1.48†	2.14 ± 1.63†‡	< 0.001
Congestive heart failure, n (%)	0 (0)	4 (12)	15 (24)	< 0.001
Hypertension, n (%)	0 (0)	18 (55)	37 (59)	< 0.001
Age ≥ 75 years, n (%)	8 (16)	7 (21)	11 (17)	0.83
Diabetes mellitus, n (%)	0 (0)	4 (12)	11 (17)	< 0.001
Dyslipidemia, n (%)	0 (0)	16 (48)	14 (22)	< 0.001
Stroke/TIA, n (%)	0 (0)	32 (97)	3 (5)	< 0.001
Peripheral artery disease, n (%)	0 (0)	2 (6)	5 (8)	0.046
eGFR (mL/min/1.73m^2^)	74 ± 23	64 ± 23*	68 ± 17	0.060
Cardiac medications				
Digitalis, n (%)	0 (0)	0 (0)	0 (0)	–
Diuretics, n (%)	0 (0)	3 (9)	10 (16)	0.013
Nitrates, n (%)	0 (0)	2 (6)	0 (0)	0.031
ARBs/ACEIs, n (%)	0 (0)	12 (36)	23 (37)	< 0.001
Calcium channel blockers, n (%)	0 (0)	11 (33)	20 (32)	< 0.001
Beta-blockers, n (%)	0 (0)	3 (9)	21 (33)	< 0.001
Antiarrhythmic drugs, n (%)	0 (0)	0 (0)	20 (32)	< 0.001

Values are mean (±SD) or number of subjects (%)

*ACEI* Indicates angiotensin converting enzyme inhibitors, *ARB* Angiotensin receptor blockers, *eGFR* Estimated glomerular filtration rate, and *TIA* transient ischemic attack

*P < 0.05 and †*P* < 0.01 vs Healthy controls; §*P* < 0.05 and ‡*P* < 0.01 vs Disease controls

Echocardiographic data are presented in Table 2. LV ejection fraction seemed to be preserved in all groups. There were no statistically significant differences in LA volume index or LV mass index between PAF patients and disease controls. Disease controls had a slight increase in E/E’, but did not seem to be in heart failure condition.

**Table 2:** Echocardiographic data of the study groups

Parameters	Healthy controls *(n = 50)*	Disease controls *(n = 33)*	PAF patients *(n = 63)*	*P*
LA diameter (mm)	32 ± 5	40 ± 7†	43 ± 7†	< 0.001
LA volume (mL)	32 ± 10	44 ± 10†	49 ± 15†	< 0.001
LA volume index (mL/m^2^)	19 ± 6	27 ± 5†	28 ± 9†	< 0.001
LV end-diastolic dimension (mm)	42 ± 5	47 ± 7†	49 ± 7†	< 0.001
LV ejection fraction (%)	65 ± 5	61 ± 12	62 ± 8	0.088
Thickness of IVS (mm)	9 ± 1	10 ± 2†	10 ± 2†	< 0.001
Thickness of posterior wall (mm)	9 ± 1	10 ± 1†	9 ± 2†§	< 0.001
LV mass (g)	120 ± 29	177 ± 65†	171 ± 65†	< 0.001
LV mass index (g/m^2^)	73 ± 14	108 ± 33†	98 ± 30†	< 0.001
LV hypertrophy (%)	0 (0)	17 (52)	17 (27)	< 0.001
E velocity (cm/s)	63 ± 13	60 ± 23	64 ± 17	0.59
A velocity (cm/s)	71 ± 20	80 ± 25	61 ± 23*‡	0.001
E/A	0.95 ± 0.33	0.83 ± 0.46*	1.21 ± 0.56*‡	< 0.001
Deceleration time (ms)	213 ± 56	224 ± 68	205 ± 70	0.41
E’, septal (ms)	7.7 ± 2.3	6.1 ± 2.5†	7.7 ± 2.4‡	< 0.001
E’, lateral (ms)	10.3 ± 3.1	7.4 ± 2.9†	9.5 ± 2.8‡	< 0.001
Averaged E’ (ms)	9.0 ± 2.5	6.7 ± 2.6†	8.6 ± 2.3‡	< 0.001
A’, septal (ms)	10.0 ± 1.7	8.8 ± 2.2*	7.6 ± 2.6†§	< 0.001
A’, lateral (ms)	10.8 ± 2.5	9.5 ± 2.6*	8.0 ± 3.0†§	< 0.001
Averaged A’ (ms)	10.4 ± 1.8	9.2 ± 2.3*	7.8 ± 2.7†§	< 0.001
Averaged E/E’,	7.4 ± 2.3	10.0 ± 5.4	7.9 ± 2.8	0.086
Averaged E’/A’	0.90 ± 0.33	0.79 ± 0.42	1.27 ± 0.63†‡	< 0.001

Values are mean (±SD) or number of subjects (%)

*IVS* Indicates interventricular septum

*P < 0.05 and †*P* < 0.01 vs Healthy controls; §P < 0.05 and ‡P < 0.01 vs Disease controls

### Comparisons of AEMD between the study groups

**Fig. 4:**
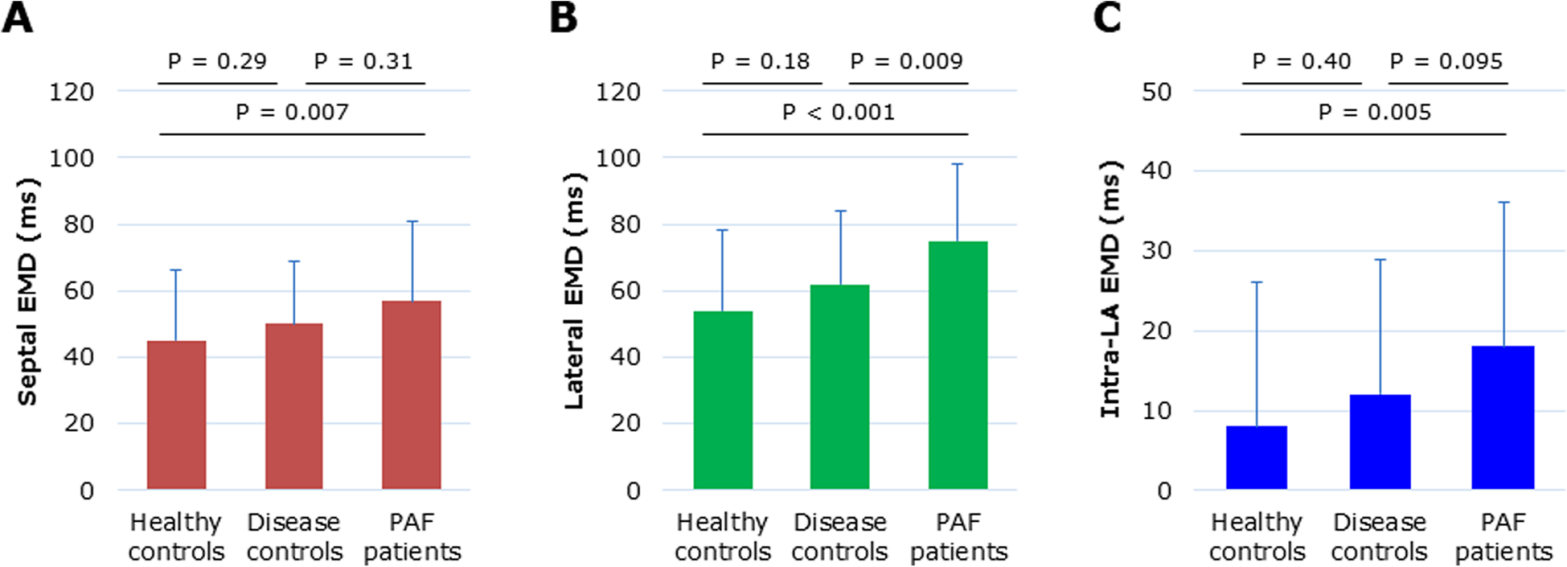
Comparisons of the septal EMD (**a**), lateral EMD (**b**), and Intra-LA EMD (**c**) between the study groups. Data are shown in mean (±SD)

Figure 4 compares AEMD-related variables for the 3 groups. PAF patients (75 ± 23 ms) had a longer period of AEMD, the lateral EMD in particular, compared with disease (62 ± 22 ms, *P* = 0.009) and healthy (54 ± 24 ms, *P* < 0.001) controls. The septal EMD and intra-LA EMD were not prolonged enough to discriminate between PAF patients and disease controls.

### Potential usefulness of AEMD for identifying PAF patients

**Fig. 5:**
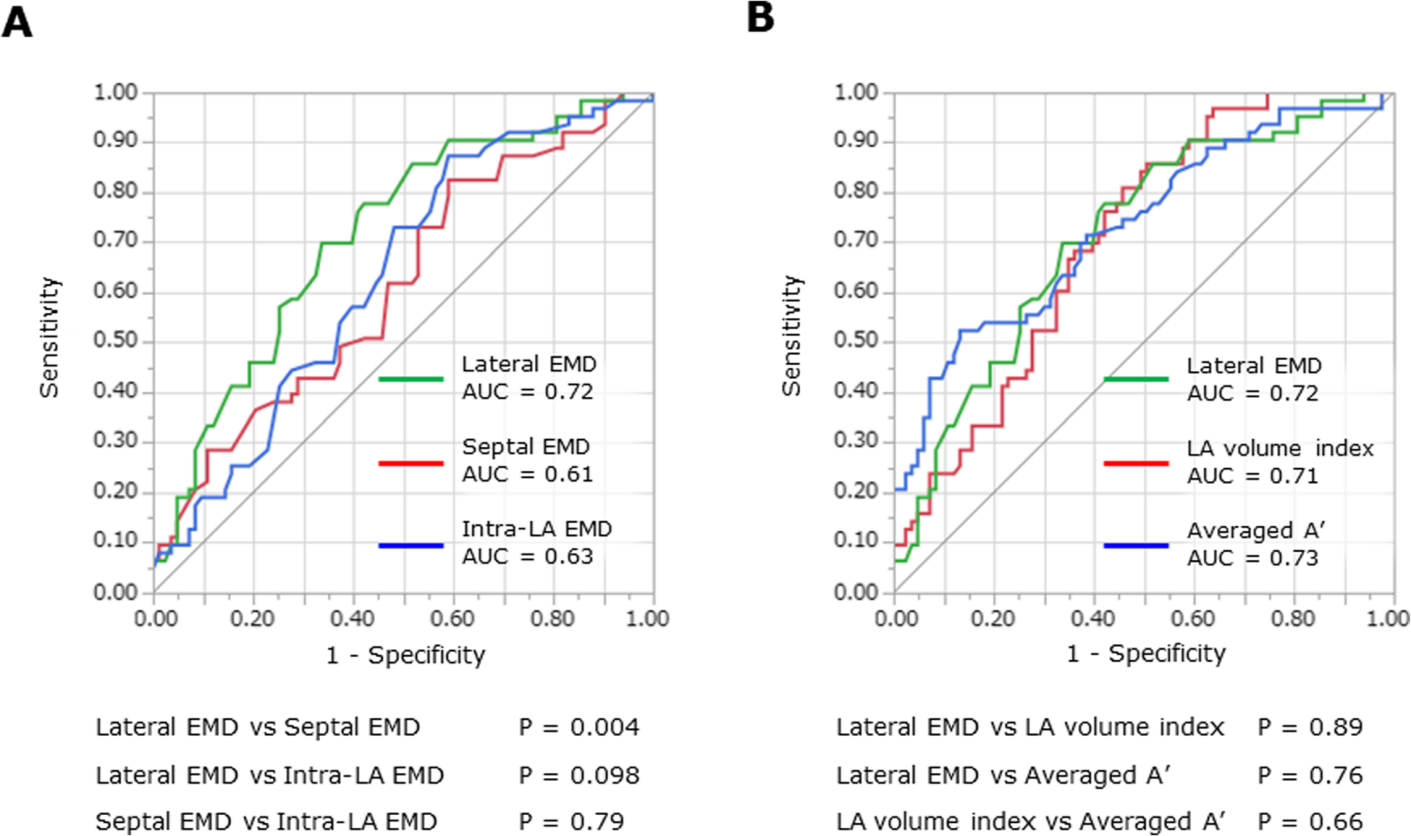
ROC analysis for identifying PAF patients, comparing AUCs of AEMD-related variables (**a**), and AUCs of the lateral EMD, LA volume index, and the averaged A’ (**b**)

For the total population (*n* = 146), usefulness of AEMD-related variables for identifying PAF patients was assessed by using ROC analysis. As shown in Fig. 5a, the lateral EMD had larger AUC compared with the septal EMD (*P* = 0.004) and intra-LA EMD (*P* = 0.098). The subsequent analysis, examining relative usefulness of the lateral EMD to LA volume index and averaged A’, showed no significant differences in AUCs of these indices (Fig. 5b). With a cut-off value of the lateral EMD set at ≥ 67 ms, the sensitivity, specificity, and positive predictive value for identifying PAF patients were 70, 66%, and 61%, respectively.

Table 3 shows the results of logistic regression analysis for identifying PAF patients. With univariate analysis, variables that significantly related to the PAF condition (*P* < 0.05) were body mass index, LA volume index, averaged A’, and the lateral EMD. Multivariate analysis revealed that the lateral EMD (OR 1.25, 95%CI 1.03–1.52, *P* = 0.023), along with LA volume index (OR 2.25, 95%CI 1.44–3.51, *P* < 0.001), was one of the significant independent associate of identifying PAF patients.

**Table 3:** Univariate and multivariate logistic regression analysis for identifying PAF patients

Parameters	Univariate analysis			Multivariate analysis		
	OR	95% CI	*P*	OR	95% CI	*P*
Age (per 10 years)	0.98	0.77–1.24	0.85	1.33	0.87–2.05	0.19
Body mass index per 5.0	2.18	1.34–3.56	0.002	1.99	1.02–3.88	0.044
CHA_2_DS_2_-VASc score	1.0	0.84–1.19	1.0	0.77	0.56–1.06	0.11
eGFR (per 10 mL/min/1.73m^2^)	0.94	0.80–1.11	0.49	1.25	0.95–1.64	0.11
LA volume index (per 5 mL/m^2^)	1.96	1.43–2.69	< 0.001	2.25	1.44–3.51	< 0.001
LV hypertrophy (= 1)	1.43	0.66–3.10	0.36	0.38	0.11–1.28	0.12
Averaged A’	0.68	0.58–0.80	< 0.001	0.68	0.55–0.84	< 0.001
Lateral EMD (per 10 ms)	1.41	1.20–1.65	< 0.001	1.25	1.03–1.52	0.023

Abbreviations are the same as in Table 1

## Discussion

The main finding of the present study was that AEMD, particularly for the lateral side, was prolonged in PAF patients compared not only with the healthy individuals but also with MRFs patients (considered to be at high-risk for AF) and that with ROC and multivariate analysis, the lateral EMD had noninferiority to LA volume index, known as a strong predictor of AF, for identifying PAF patients.

### Previous studies on AEMD and AF

There are several reports on the relationship between AEMD and AF. Ari et al. initially reported that in 50 patients with persistent AF who underwent successful electrical cardioversion, a relatively short AEMD was associated with maintained sinus rhythm at 1-month follow-up [7]. They also found that the lateral EMD was one of the significant predictors of AF recurrence, along with LA volume index and LV inflow A wave velocity [7]. In 108 PAF patients and 52 healthy controls, Hoshi et al. found that prolonged AEMD (81%) was frequently associated with PAF, the percentage of which was greater than LA volume index (53%) and lateral A’ (52%) [8]. Results of ours were consistent with, and supportive of, those in these previous studies [7, 8]. Among patients with prior ischemic stroke, the clinical background similar to our MRFs patients, AEMD was shown to be prolonged (not as much prolonged as in AF patients) compared with normal controls [13].

### Potential mechanisms of prolonged AEMD in AF

Longer AEMD implies more heterogeneous propagation impulse within the atria that may be compromised by fatty replacement [17]. Although no experimental evidence has existed on this assumption, prolonged atrial conduction time is found to be associated with increased LA diameter and reduced LA systolic function, supporting the notion that AEMD reflects structural and electrophysiological remodeling of the atrium [18–20]. Another reason for the relatively prolonged AEMD in PAF patients may be related to an effect of inflammatory cytokines [21]. Systemic inflammation reportedly causes subclinical cardiac damage even in an early phase of atherosclerosis [22]. Some disorders in which inflammation underlies, such as diabetes mellitus and ulcerative colitis, are shown to be associated with AEMD prolongation [9–11].

Nevertheless, not all PAF patients were considered to have significant atrial involvement with irreversible atrial mechanical function. Because of the paroxysmal nature, some patients might have atrial mechanical function halfway recovered at the time of echocardiography. This may be supported by the finding that in PAF patients, E/e’ was not as high as expected while their LV filling pattern showed “restrictive” [23]. Whether AEMD shortens concurrently with improved atrial mechanical function over time awaits further investigations.

In the present study, only the lateral EMD emerged as a significant predictor of identifying PAF patients. From a histopathological viewpoint, myocytes of the left atrium are irregularly arranged compared with those of the right atrium [24]. Given that degenerated atrial tissue is associated with atrial current running in a non-uniform manner [25], AEMD prolongation greater at the lateral side compared with the septal is plausible, further suggesting that the lateral EMD is more likely related to AF vulnerability.

### Clinical implications

To the best of our knowledge, this is the first report describing the significance of AEMD that is analyzed along with “disease controls”. One important finding from our results is that AEMD was prolonged in PAF patients compared with MRFs patients who had a certain degree of LA enlargement and diastolic dysfunction. Underlying mechanisms for the different AEMD between the groups despite similar clinical and functional features remain unclear, but may deserve to be addressed with further investigations.

### Limitations

The single-center, cross-sectional study was an inherent limitation in this study. We used “PAF” as a surrogate for AF prediction or recurrence and thus our results cannot be extrapolated to other situations. Follow-up examinations were not performed. This was because in addition to the small number of patients, a certain number of patients had experienced stroke or received anti-arrhythmic drugs, which might preclude meaningful results that would be drawn. Another limitation was that the possibility of occult PAF occurring among patients in disease controls could not be excluded. However, no patients with MRFs had been reported to have AF during hospitalization, to have intracardiac thrombus, or to ever be anticoagulated. Other variables that might have related to AF were not available, such as the right-sided AEMD or a novel index of LA strain [26]. Finally, influence of anti-arrhythmic drugs on AEMD remains to be evaluated.

## Conclusions

The preset study evaluated usefulness of AEMD for identifying PAF patients. We found that AEMD, particularly for the lateral side, was prolonged to a more extent in PAF patients compared with healthy subjects in addition to MRFs patients. Also, AEMD was shown to have noninferiority to LA volume index in identifying PAF patients. Prospective studies, with a larger number of subjects, are needed to confirm our results and to identify thresholds at which any abnormal values of AEMD alter clinical management in patients with various cardiovascular conditions.

## Data Availability

All data generated or analyzed during this study are included in this published article [and its supplementary information files].

## Abbreviations

AEMD: Atrial electromechanical delay
AF: Atrial fibrillation
AUC: Area under curve
EMD: Electromechanical delay
LA: Left atrial
LV: Left ventricular
MRFs: Multiple cardiovascular risk factors
PAF: Paroxysmal atrial fibrillation
ROC: Receiver operating characteristic
